# Assessing cortisol from hair samples in a large observational cohort: The Whitehall II study

**DOI:** 10.1016/j.psyneuen.2016.07.214

**Published:** 2016-11

**Authors:** Jessica G. Abell, Tobias Stalder, Jane E. Ferrie, Martin J. Shipley, Clemens Kirschbaum, Mika Kivimäki, Meena Kumari

**Affiliations:** a University College London, Department of Epidemiology and Public Health, London, UK; b Department of Psychology, TU Dresden, Dresden, Germany; c School of Social and Community Medicine, University of Bristol, UK; d Institute for Social and Economic Research, University of Essex, UK

**Keywords:** Cortisol, Depressive symptoms, Diabetes, Medications

## Abstract

Hair cortisol concentrations (HCC) have been suggested to reflect long-term integrated cortisol levels, but most evidence of associations with co-variates is from small samples of healthy volunteers.

The objective of this study was to describe the collection of hair samples in a large cohort study and report associations of demographic and health measures with HCC. We examined HCC measured from the 3 cm hair segment near the scalp in 3507 participants (aged 59–83 y) from The Whitehall II occupational cohort study of British civil servants. Hair samples were analysed using a column switching LC–APCI–MS/MS assay. Findings from mutually adjusted linear regression analyses revealed lower HCC in participants who reported use of hair dye [% difference (95%CI); −12.5 (−22.0, −1.9), p value = 0.022] and evidence suggestive of differences by length of sample storage and seasonal variation. With regard to demographic variables, HCC was lower in women compared to men [−17.0 (−24.8, −8.4), p value <0.001] and higher in Black compared to other ethnic groups. Prevalent diabetes, use of systemic corticosteroids and cardiovascular medication were independently associated with higher HCC. With regard to health, depressive symptoms were associated with higher HCC [20.0 (8.1, 33.3), p value = 0.001] following adjustment for physical disease and medication. We conclude that hair steroid analysis presents significant opportunities for assessing cortisol in large scale cohorts. Demographic factors, sample storage, season of collection and hair characteristics should be considered in future analyses. Health status, both mental and physical, is linked to HCC.

## Highlights

• Hair samples present considerable opportunities for assessing cortisol in cohorts.
• Certain hair characteristics correlate with hair cortisol concentrations (HCC).
• Mental and physical health status is independently associated with HCC.

## 1 Introduction

Understanding the role of stress in health continues to be a major aim in epidemiological research. Activation of the hypothalamic-pituitary-adrenal (HPA) axis is a primary response to a stressful stimulus and cortisol, the end product of the HPA axis, which may mark exposure to chronic stress, is proposed as a mediator between adverse social conditions and physical health.

Observational surveys frequently collect cortisol from their participants to allow these pathways between stress and health to be examined. Traditionally, the assessment of cortisol in observational surveys has been through measurements in blood, saliva or urine. However, high state reactivity and the pulsatile secretion of cortisol (Hellhammer et al., 2007, Lightman et al., 2008, Young et al., 2004) mean single measurements reflect short-term levels, ranging from minutes (plasma or saliva) to hours (urine), and provide limited information about long-term cortisol secretion. Repeated ambulatory measurements which capture longer-term levels, are time-consuming, expensive and may include high proportions of missing data due to participant non-compliance (Broderick et al., 2004). In addition, these methods are often invasive and require a clinic environment to aid collection or they require high levels of commitment from the participants, which becomes harder to request in certain groups e.g. studies of ageing. Thus, blood, saliva and urine measures, while well-suited for capturing dynamic aspects of endocrine activity, (e.g., acute stress reactivity or diurnal rhythmicity), are less useful as markers of the long-term secretory patterns. However, long-term cortisol levels may be important in the aetiology of chronic disease, since the cumulative burden of frequent or enduring HPA axis activation may be associated with a range of maladaptive effects on the organism.

Hair cortisol concentration (HCC) can provide an alternative assessment method, capturing information about cortisol levels over the course of several months. The collection of hair samples is non-invasive and can be performed by non-health care workers at any time of the day. Hair samples can also be stored at room temperature and be sent by mail. Although forensic science and toxicology have an established history of analysing hair for exogenous substances, recent reports that endogenous steroids can also be detected in hair have expanded research using human hair samples (Meyer and Novak, 2012, Russell et al., 2012, Stalder and Kirschbaum, 2012). Direct and in-direct validation studies have confirmed the capacity of these measures to provide an index of integrated long-term cortisol secretion (Meyer and Novak, 2012, Russell et al., 2012, Stalder and Kirschbaum, 2012) despite lack of understanding of the precise means by which cortisol is incorporated into the hair shaft (Sharpley et al., 2012). High intra-individual stability in HCC supports the use of this method in epidemiological research to assess long- term cortisol secretion (Stalder et al., 2012b).

Several issues potentially affect HCC measurements. First, small scale studies have documented differences in HCC by a range of characteristics such as sex, age and ethnicity implying that without controlling for these and other confounding factors, biased results could be obtained (Russell et al., 2012, Sharpley et al., 2012, Staufenbiel et al., 2015, Wosu et al., 2013). Second, differences in HCC by physical and mental health have been observed, with elevated HCC in patients with chronic conditions such as diabetes (Feller et al., 2014, Henley et al., 2013, Manenschijn et al., 2013) and myocardial infarction (Pereg et al., 2011) as well as mental health problems (Staufenbiel et al., 2013). These findings suggest that when measuring HCC it may be important to take into account health status, although these studies were often composed of small samples of patients or based on self-reported diagnoses and therefore the exact effect on HCC remains unclear. Third, human hair is exposed to exogenous factors, including hair maintenance products, such as hair dye, shampoo and chemical treatments. Identifying whether these factors are potential sources of measurement error is crucial (Dettenborn et al., 2012b, Feller et al., 2014, Wosu et al., 2015). Although certain characteristics, such as dyeing and other chemical treatment of hair have been found to be associated with reduced HCC (Manenschijn et al., 2011, Stalder et al., 2013), samples have not always been large enough to examine the full range of hair maintenance procedures.

To date, HCC has been assessed in some larger samples up to N = 1258 (Braig et al., 2015, Feller et al., 2014, Stalder et al., 2013, Staufenbiel et al., 2015) but the majority of them may lack representiveness, being drawn from volunteers. To address limitations of sample size and generalisability we assessed HCC in a large well-characterised longitudinal cohort study to examine the feasibility of this measure for future observational research. Our objectives were to describe the procedure of collecting and validating hair samples for HCC analysis and to examine the association between HCC and a range of factors. We examined these data in relation to hair characteristics and demographical characteristics. We also explored the influence of sample storage and seasonal effects, the impact of which are not well known. Furthermore, we wished to examine the association between clinically screened physical health events and mental health status with HCC.

## 2 Methods

### 2.1 Study population

The Whitehall II study is an occupational cohort originally recruited from London based Civil Service departments in 1985–1988 (phase 1). The initial sample consisted of 10,308 participants who undertook a clinic screening and completed a postal questionnaire. Follow up screening examinations were conducted in 1991–1993 (phase 3) and 1997–1999 (phase 5), 2003–2004 (phase 7), 2007–2009 (phase 9) and 2012–2013 (phase 11). Postal questionnaires were sent to participants in 1989 (phase 2), 1995 (phase 4), 2001 (phase 6) and 2006 (phase 8). Hair cortisol data were collected for the first time in 2012–2013 (phase 11). Further details of the Whitehall II Study can be found elsewhere (Marmot and Brunner, 2005). Ethical approval for the Whitehall II study was obtained from the University College London Medical School committee on the ethics of human research.

### 2.2 Hair sample collection

A total of 6,308 participants were included in phase 11, of whom 5,660 undertook a clinical screening. A sample of hair was taken from 4,460 participants, excluding those who had insufficient hair (N = 997), who had a severe head tremor (N = 31) or who didn’t consent (N = 172). Women were more likely to provide a sample than men (78.6% vs. 67.4%). Hair strands taken from the head by a nurse were cut carefully with scissors as close as possible to the scalp. Hair was collected from the *vertex posterior* region of the head, since it has been found that this area of the scalp has the greatest growth cycle synchrony and exhibits the lowest intra-individual variability in HCC (Sauve et al., 2007). Steroid concentrations were determined from the 3 cm segment of hair closest to the scalp. This represents hair growth over the three month period prior to sampling based on an average hair growth of 1 cm/month (Wennig, 2000).

### 2.3 Sample preparation

The samples were analysed between 23rd June and 20th August in 2014 using a column switching LC–APCI–MS/MS assay, which has been found to be a sensitive, reliable method for quantifying steroids in human hair (Gao et al., 2013). The samples were washed and steroids extracted following the protocol described previously (Gao et al., 2013, Stalder et al., 2012b) with minor changes to allow analysis by liquid chromatography-tandem mass spectrometry (LC–MS/MS). The intra and inter-assay coefficients of variation (CVs) for cortisol analysis by this method have been reported to range between 3.7% and 8.8%. 11 samples were not analysed due to insufficient sample or technical error. A total of 4449 samples were analysed for HCC but samples that weighed less than 7.5 mg (N = 404) were excluded from the analyses for this study as were those with samples shorter than 3 cm (N = 61). Following sensitivity analysis, samples shorter than 3 cm (N = 193) but with sufficient weight were included, since shorter length alone was not associated with differences in HCC. Samples in which the scalp end of the hair was not obvious were also excluded (N = 7). This left a total of 3,977 respondents, of whom 3,966 had also completed the accompanying hair questionnaire.

### 2.4 Assessment of covariates

Information about hair-related characteristics was collected via a questionnaire administered when the hair sample was collected. Participants were asked whether they dyed or treated (perm or chemical treatment) their hair. They were also asked about the number of times per week they washed their hair with shampoo. This was recoded as: once a week or less, 2–4 times per week and daily or 5–6 times per week. Interviewers’ assessed the colour of the hair sample taken, although participants were also asked about their natural hair colour, if they had used hair dye. Seven categories of hair colour were specified on the questionnaire (1: grey/white, 2: blond, 3: red, 4: brown, 5: black, 6: don’t know, 7: other). These were recoded into five categories: (1) grey/white, (2) blonde (3) red, (4) brown, (5) black. If ‘don’t know’ was specified then this was recoded into missing. If ‘other’ was chosen and another hair colour was specified in the open question provided, these were recoded.

Information on age, sex, last-known civil service employment grade and ethnicity was obtained from the general questionnaire at phase 11. Civil service employment grade was defined using three tiers: (1) administrative, (2) professional or executive and (3) clerical or support. Ethnicity was self-defined as White, South Asian, Black or Other. Participants provided details of current medications use (generic name, brand name, or both); these were subsequently coded using the British National Formulary. This study used information about participants who used steroid medication (local and systemic) and cardiovascular medication in the last 14 days. Date of hair collection was used to calculate variables related to length of sample storage and season of collection: winter (Dec, Jan, Feb), spring (Mar, Apr, May), summer (Jun.–Aug.) and autumn (Sep.–Nov.). An eight category variable was created to take account of hair growth which during the three month sample overlapped two seasons: (1: winter, 2: winter-spring, 3: spring, 4: spring-summer, 5: summer, 6: summer-autumn, 7: autumn, 8: autumn-winter). Mental health status was assessed using The Center for Epidemiologic Studies–Depression scale (CES–D), a 20 item scale that measures symptoms of depression in the general public, a score of 16+ has previously been used to classify depression (Radloff, 1977). Height and weight were measured using standardised protocols during the medical examination and BMI (kg/m^2^) was calculated. Participants were asked about doctor diagnosis of diabetes at each phase. Diabetes medication was extracted, again using British National Formulary codes (BNF). Venous blood was taken at study phases 3, 5, 7, 9 and 11 to assess biomarkers, such as fasting glucose and HbA1c (plasma cortisol was not measured). Prevalent diabetes at phase 11 was defined either by fasting glucose (fasting ≥7) (WHO, 2006) or HbA1c (≥6.5%) (WHO, 2011) or self-reported diabetes; doctor diagnosis, use of insulin or oral glucose lowering drugs. Prevalent CHD was assessed from medical records 1985–2009, supplemented by reports of doctor diagnosis at phase 11 and self-reports of a heart attack, angina pectoris or other heart trouble since 2008. Stroke, likewise was assessed using medical records up to phase 9 or doctor diagnosis/self-report of a stroke since 2008.

### 2.5 Statistical analysis

HCC values were positively skewed and therefore log transformed to establish a normal distribution. Eighty-five outliers ±3SD from the mean were excluded from the analyses. The association between each exposure variable (independent variable) and HCC was assessed initially using linear regression and subsequently using multiple regression with adjustment for potential confounding variables. Regression analyses were first conducted on demographics and hair related variables, followed by medication and health status. Percentage differences in mean HCC between the levels of each exposure variable were calculated from the regression coefficients for each exposure variable using the formula: Percentage difference = (exp(coefficient) − 1.0) × 100.

## 3 Results

Table 1 provides characteristics of the participants included in the final sample. This analysis was based on 3675 participants who provided a hair sample of sufficient weight and an identifiable scalp end, completed a hair questionnaire and did not have missing data on the covariates of interest. Mean age of participants at phase 11 was 70 years. The majority of participants were men (68.4%), reported their ethnicity as white (94.2%) and had a last known civil service employment grade of executive or above. Sample collection was distributed evenly across the year and most had been stored for more than 18 months before analysis, because samples were assayed in a single run at the end of data collection. Seven percent of participants (N = 264) were taking local corticosteroids and 2% (N = 70) systemic corticosteroids. The majority of the participants from whom samples were collected were free of chronic health conditions and had a BMI between 18.5 and 29.9. Table 2 shows the characteristics of those included and not included in this analysis. Overall the samples were similar across a range of variables, although differences by sex and mean HCC were observed.

Table 3 shows that women had lower HCC than men both before and after adjustment for potential confounders. There was no significant difference in HCC by age group or when age was used as a continuous variable (percentage difference per year of age = 0.1%, p = 0.73). There was no initial difference in HCC by ethnic group, however in mutually adjusted analysis those who identified as Black had nearly 40% higher HCC than White groups. In the initial simple linear regression analysis, HCC was lower in the lower employment grades (prof/exec clerical/support) than in the higher (administrative) employment grade, but this association attenuated following adjustment.

Those who reported having dyed hair had lower HCC than those who did not. Although those who reported chemically treated hair had higher HCC initially, most of this difference was removed after adjustment for demographic and other hair characteristics. There was no significant difference in HCC by frequency of hair washing in the linear model. However, after controlling for other hair characteristics those who washed their hair more than 4 times a week had lower HCC than those who only washed it once a week or less, although this was not statistically significant (p = 0.08). The mutually adjusted analysis showed that those with black hair had slightly higher HCC on average, compared to those who had grey or white hair.

Hair samples with hair growth from winter-spring, spring, spring-summer, summer and summer-autumn had higher HCC compared to those collected in winter, but these differences remained for the winter-spring, spring, spring-summer and summer, seasons only in the adjusted analyses. HCC was lower in hair samples stored for over 18 months, compared to those stored for <18 months. A negative association (r = −0.02, p = 0.001) between months of storage and HCC was also observed when months of storage was included as a continuous variable in unadjusted linear analysis. The large difference observed between unadjusted and adjusted analyses for months of storage is perhaps due to the correlation between storage and season e.g. no sample collected in Spring in the study was stored for <18 months.

**Fig. 1:**
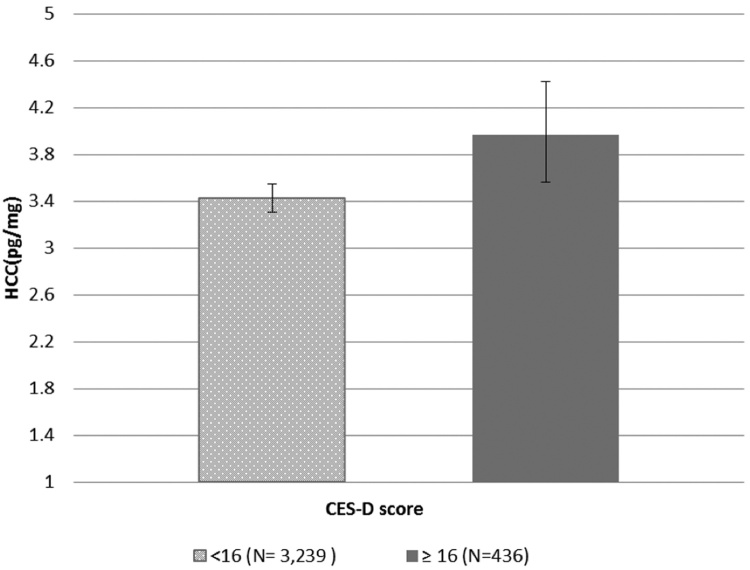
Geometric mean (±95% CI) Hair Cortisol Concentrations in participants by CES-D depressive symptoms scale ^a^. ^a^P value from *t*-test = 0.01.

Table 4 presents the association of HCC with medication intake and health status. Participants who took systemic corticosteroids had lower HCC compared to those who did not and this association remained following adjustment for demographic factors, hair characteristics and health status. Those who were currently taking CVD medication compared to those who were not, had higher HCC initially, although this association was no longer significant in adjusted analysis. There was a significant association between BMI and HCC, participants who had a BMI higher than 25 kg/m^2^ (healthy weight) had higher HCC. Participants with diabetes exhibited higher HCC than those who did not and this remained significant in the fully adjusted analysis. HCC was unrelated to CHD diagnosis or the experience of a stroke. A positive association between HCC and depressive symptoms was observed in the simple linear regression which remained when other factors were taken into account (Fig. 1).

## 4 Discussion

This study is the first to test the feasibility of using of hair cortisol as a measure of long-term cortisol exposure in a large scale occupational cohort study. Sample collection was well tolerated, quick and relatively cheap. The study was well-powered to detect correlates of HCC and our results show associations of HCC with hair treatment, hair washing, season of sample collection and health conditions.

We considered a group of hair specific factors, collected in the accompanying questionnaire and which have been considered previously. Our results suggest those participants who had black hair having slightly higher HCC than those with grey/white hair. This contrasts with previous research which suggests no influence of natural hair colour on HCC in human hair samples (Dettenborn et al., 2012a, Dettenborn et al., 2012b, Kirschbaum et al., 2009, Manenschijn et al., 2011, Raul et al., 2004, Sauve et al., 2007). The majority of previous studies have not distinguished between hair treatment and hair dye. Those studies which did, found participants who had dyed their hair had lower HCC, in accord with our results (Manenschijn et al., 2011, Sauve et al., 2007). Studies which did not differentiate between chemical treatment and hair dye also found lower HCC in participants who reported treating their hair (Manenschijn et al., 2012, Stalder et al., 2013). In our study, we found that hair treatments, such as perming or straightening, did not have an independent association with HCC after adjusting for the presence of hair dye. We see a consistent association between hair dye and HCC and therefore recommend that information about whether participants use hair dye is always collected and included in HCC analysis.

We found that participants who washed their hair every day had lower HCC than those once a week or less, although this effect was relatively small. This finding stands in contrast to most previous studies which have found no significant relationship between HCC and regular hair washing with shampoo (Groeneveld et al., 2013, Kirschbaum et al., 2009, Manenschijn et al., 2011, Stalder et al., 2013, Stalder et al., 2010). An experimental study found HCC decreased after washing with shampoo (Li et al., 2012), although this was only based on a sample size of three and hair samples were immersed in a shampoo solution. Dettenborn et al., 2012a, Dettenborn et al., 2012b found a negative association between hair washing frequency and HCC, but only in the hair segment most distal from the scalp (Dettenborn et al., 2012a). Although an association between frequency of hair washing and HCC is suggested, this does not remain in mutually adjusted analysis. We recommend that studies collect information about the frequency of hair washing and additional research explores this issue further.

Seasonal variation has been examined previously, with higher HCC reported in the summer months and lower HCC in winter (Braig et al., 2015, Staufenbiel et al., 2015). The present results based on a large sample and even sample collection across the four seasons suggested seasonal differences in HCC in the opposite direction, i.e. we found *lower* HCC in samples collected in winter-spring, spring, spring-summer and summer seasons compared to those collected in the winter. This could suggest seasonal differences in environmental influences on hair samples or seasonal variation in long-term cortisol secretion. Several hypotheses for the influence of season on HCC have been proposed, including temperature, fluid ingestion and transpiration (Braig et al., 2015). Seasonal variation in mood could also be a confounding factor, although, in our sample the association between HCC and season was unaffected when adjustment was made for depressive symptoms. A possible interaction between season of sample collection and length of storage has also been suggested (Braig et al., 2015) although few studies have examined the influence of length of storage on HCC. We found a storage effect, with HCC lower in samples that had been stored for longer. Importantly, our results suggest that the respective influences of the season of hair collection and of sample storage are independent of each other, i.e. they emerged after mutual accounting for each other. There are a number of reasons which might explain why previous findings on the seasonal variation in cortisol levels are inconsistent. Firstly the previous studies have used samples drawn from different populations from different European countries: Braig et al. (2015) examined the association between season and HCC in a sample of mothers who had recently given birth in Germany, whilst Staufenbiel and colleagues examined HCC in healthy, middle-aged women and men in the Netherlands (2015). Although these are both European countries, it is possible that temperature and humidity levels may differ between these countries and the UK. Furthermore, it is not possible to completely rule out an interaction between season and storage in earlier studies. Since in large scale observational studies it will not always be possible to control the season which sample collection occurs in or the length of sample storage, it is advisable to collect sufficient information to allow these environmental features to be taken into account.

Sex remained a significant predictor of HCC in the present study after adjustment for a range of other characteristics, with women having lower HCC than men. Overall results from previous work on sex differences in HCC have been mixed. A recent study found that sex differences were not independent of other covariates (Feller et al., 2014) and others have found no differences (Gao et al., 2010, Manenschijn et al., 2011, Raul et al., 2004, Stalder et al., 2013, Thomson et al., 2010). However, Dettenborn et al., 2012a, Dettenborn et al., 2012b also found elevated HCC in men (Dettenborn et al., 2012b). This accords with the present findings and also two previous studies, which demonstrate higher HCC in males compared to females (Manenschijn et al., 2013, Staufenbiel et al., 2015). A possible mechanism for higher HCC in men compared to women is the higher 24-h urinary glucocorticoid secretion rate in men (Remer et al., 2008).

In common with the majority of previous studies (Kirschbaum et al., 2009, Manenschijn et al., 2011, Manenschijn et al., 2012, Raul et al., 2004, Stalder et al., 2012a), we found no association between HCC and age in our sample of participants aged 59–84. However, research that has examined individuals across wider age ranges reported positive associations between age and HCC (Dettenborn et al., 2012b, Feller et al., 2014, Stalder et al., 2013), with HCC increased for adults between 50 and 91 years. Although these differences may be due to the different samples under observation, this may also suggest that such age-related changes in HCC occur in early old age. A recent study which specifically examined HCC in adults between 47 and 82 years found age-related changes in HCC, which were consistent even after controlling for other factors (Feller et al., 2014). However, strong positive correlations have also been observed between age and HCC in studies with narrower age ranges (Manenschijn et al., 2013, Staufenbiel et al., 2015).

Several hypotheses have been offered to explain a difference in HCC by ethnicity (Wosu et al., 2015, Wosu et al., 2013). Firstly, that certain ethnic groups are more likely to be exposed to social stressors, secondly that there are differences in the hair texture and growth rate in different ethnic groups and thirdly that hair maintenance practices may differ amongst ethnic groups. Our findings suggest higher HCC in Black groups compared to other ethnic groups. Studies from the US have reported no differences by ethnicity and higher HCC in Black groups (Wosu et al., 2015) (O'Brien et al., 2013), however both have suggested that their findings might be confounded by socioeconomic position. We found that this association was independent of last known employment grade, suggesting that socioeconomic position might not fully explain these disparities. We also found these differences by ethnicity to be independent of washing frequency, use of hair dye and chemical treatment which suggests that hair maintenance practices may not account for these differences either. However, these findings require further analyses because of the small number of cases of each different ethnic group in our population.

In our study an initial difference in HCC by last known employment grade was found. However, this association was not independent of sex. Several articles have examined the association between educational level and HCC in adults (Boesch et al., 2015, Serwinski et al., 2016) and children (Rippe et al., 2016). Chen et al. (2013) found no association between educational level and HCC in adults in China. At the current screening many participants were retired, therefore these analyses need further exploration with non-occupational measures of social position.

To the best of our knowledge, the association of medication intake with hair cortisol levels has not been examined, although a study which examined general medication intake found no difference in HCC (Kirschbaum et al., 2009). Due to the size and structure of our cohort we were able to examine the association between HCC and several groups of medication. We found that those who took systemic corticosteroids had lower HCC compared to those who took no steroid medication. However, in a sample which retained outliers, both local and systemic were associated with higher mean HCC than those who did not (data not shown). We anticipate that certain outliers for the topical corticosteroids might be explained by participants using creams/gels that came into direct contact with the scalp/hair. Synthetic GCs (Dexamethasone, Betamethasone, etc.) should not produce a cortisol-like signal in the MS (i.e. cross-react), only the use of actual hydrocortisone medication would do this. In further analysis (Supplementary Table S1) we show that participants with lower hair cortisol were using glucocorticoid therapy and those with higher hair cortisol were using topical corticosteroids. This suggests that perhaps some of the extreme HCC results observed can be attributed to medication use. Research which has examined HCC thus far tends to exclude participants taking corticosteroid medication from the study and this seems to be a sensible strategy. We also found evidence of an association between BMI and HCC, with participants with a BMI 25 kg/m^2^ or higher (overweight or obese) having higher mean HCC than their normal weight counterparts. This finding accords with previous research which finds an association of obesity with greater cortisol excretion in urine, which also reflects overall output (Brunner et al., 2002) and studies that examine HCC and obesity in children (Veldhorst et al., 2014) and adults (Feller et al., 2014, Stalder et al., 2013, Stalder et al., 2012a). Cortisol might be directly related to adiposity in humans through adipocytes. Visceral adipose tissue expresses high concentrations of glucocorticoid receptors (Pou et al., 2007) and it is thought that adipocytes are a source of cortisol. In humans increased 11β-HSD1 activity, which regulates glucocorticoid metabolism at the tissue level, has also been associated with features of the metabolic syndrome (Walker and Andrew, 2006).

We also examined the association of HCC with three chronic conditions; diabetes, CHD and stroke. The present results revealed increased HCC in people with type 2 diabetes after full adjustment for potential confounding influences. This concurs with previous data of elevated HCC in patients with diabetes (Feller et al., 2014, Henley et al., 2013, Manenschijn et al., 2013) and is also in line with findings that HCC is positively related to glycated haemoglobin (a marker of long-term plasma glucose concentrations) and prevalence of the metabolic syndrome (Stalder et al., 2013). Despite previous research suggesting an independent relationship between HCC and cardiovascular disease (Manenschijn et al., 2013) and elevated HCC in patients with acute myocardial infarction (Pereg et al., 2011) we found no evidence of this in our cohort. Although we find an association between taking CVD medication and HCC, this does not remain when other factors, such as BMI, were taken into account. Certain CVD medications, e.g. statins, are prescribed in high-risk disease-free groups for primary prevention in addition to being prescribed as therapy for CHD patients. Certain CVD risk factors are considered by family doctors (GPs) in the UK as predictive of developing CVD at some point in the next ten years. HCC may be associated with these risk factors and predictive of being prescribed CVD medication rather than being actual markers of CHD or stroke. We have provided further information (Supplementary Table S2) in Appendix A to demonstrate this. Furthermore, it is possible that. statin use is causing weight gain (Swerdlow et al., 2015) which is then associated with HCC (p. 6).

We found an independent association between depressive symptoms, measured using the CES-D scale and HCC, such that those who reported more depressive symptoms had increased HCC. This accords with previous findings (Dettenborn et al., 2012a, Stalder et al., 2014), although most work in this area has been on small samples recruited because of their diagnosis or an existing physical health condition (Staufenbiel et al., 2013). Therefore, we believe this to be the first report of an independent association between depressive symptoms and HCC in a large observational cohort.

There are certain limitations that must be acknowledged. At this phase (11) of the study, only hair cortisol data were available, thus the HCC results presented here could not be compared to those from other types of body fluids. Since this field of research is in its infancy, a clear distribution of potential values has not been established, although this is perhaps comparable to other existing methods of cortisol assessment, e.g. in saliva or urine. Due to the age and demographic distribution of this cohort, a number of participants were not able to provide a hair sample of sufficient length for analysis. Although we attempted to minimise within individual variation in hair growth by taking hair from the vertex posterior region of the head, hair growth rates may vary between individuals. Furthermore the participants in this cohort at this phase have a mean age of 70 years older, therefore it may not possible to generalise the findings of this study to younger adults. In the Whitehall II cohort there are few participants who report that they are from an ethnic group other than white, therefore our finding on the association between ethnicity and HCC is not generalizable to a wider population and additional research will be required to explore this further. There are, however, strengths to this study, primarily that hair samples have been drawn from a large sample, from which a range of other variables have also been assessed. In addition, the quantification of HCC by LC–MS/MS, which is considered the current gold-standard approach for such analyses, comprises a clear strength of this paper.

Collecting hair samples in occupational cohorts present significant opportunities for assessing cortisol and other steroids; especially since the collection is cheaper and less invasive than previous methods of measuring cortisol. We conclude that several demographic factors, hair characteristics and details of hair care routines are correlated with hair cortisol and are important to collect when wishing to analyse cortisol in hair. We recommend that information on medication and seasonal variation should be collected and considered as possible confounders in analyses. We also recommend that further research assesses the impact of storage length on hair samples and that information regarding storage length be recorded and taken into account in the analysis. Similarly, mental and physical health is associated with HCC levels and should be taken into in the analysis. We therefore recommend collecting hair samples for assessing HCC if long-term levels of cortisol are of interest and a high response rate is unlikely using traditional repeated urine or saliva measures.

## Disclosure statement

The authors have nothing to disclose.

## Author contributions

JA and MKu designed the study and wrote the first draft of the manuscript. TS and CK conducted the sample analyses. JA analysed the data. All authors (JA, TS, JF, MJS, CK, MKi & MKu) interpreted the results and assisted with the preparation of the manuscript.

## Role of funding source

The Whitehall II study has been supported by grants from the Medical Research Council (K013351); British Heart Foundation; National Heart Lung and Blood Institute (R01HL36310), US, NIH: National Institute on Aging (R01AG13196 and R01AG34454), US, NIH; Agency for Health Care Policy Research (HS06516); and the Dunhill Medical Trust (R247/0512), UK. The collection of the hair samples was also funded by the Dunhill Medical Trust (R247/0512). MKi is supported by the Medical Research Council (K013351), Academy of Finland and an ESRC professorship. MJS is partly supported by the British Heart Foundation. MKu is partly supported by the Economic and Social Research Council (RES-596-28-0001).

## Figures and Tables

**Table 1:** Characteristics of study participants (N = 3675).

Characteristic	N	%	Median HCC
Sex:			
Male	2513	68.4	3.1
Female	1162	31.6	2.4
Age groups:			
59–64	932	25.4	2.9
65–74	1899	51.7	2.8
75–85	844	23.0	2.8
Ethnicity:			
White	3460	94.2	2.9
South Asian	151	4.1	2.5
Black	36	1.0	5.3
Other	28	0.8	4.0
Grade:			
Administrative	1811	49.3	3.0
Prof/exec	1529	41.6	2.8
Clerical/support	335	9.1	2.4
Original hair colour:			
Grey/white	2876	78.3	2.9
Blonde	96	2.6	2.4
Red	32	0.9	2.9
Brown	473	12.9	2.8
Black	198	5.4	3.1
Hair dyed:			
Yes	608	16.5	2.3
No	3067	83.5	3.0
Hair Treated:			
Yes	149	4.1	2.6
No	3526	96.0	2.9
Hair washing freq.:			
≤Once a week	1119	30.5	2.9
2–4 times per week	1716	46.7	2.8
>4 times per week	840	22.9	2.9
Season collected:			
Winter	454	12.4	3.5
Winter-spring	561	15.3	2.6
Spring	320	8.7	2.2
Spring-summer	690	18.8	2.6
Summer	337	9.2	2.8
Summer-autumn	507	13.8	2.9
Autumn	332	9.0	3.1
Autumn-winter	474	12.9	3.2
Storage (months):			
18 months or less	570	15.5	3.1
19–24 months	1557	42.4	3.2
>24 months	1548	42.1	2.5
Steroids medication:			
No	3341	90.9	2.9
Yes (local)	264	7.2	2.9
Yes (systemic)	70	1.9	1.6
CVD drugs:			
Yes	2174	59.2	2.9
No	1501	40.8	2.8
Diabetes:			
No	3166	85.7	2.8
Yes	527	14.3	3.2
CHD:			
No	2664	72.5	2.8
Yes	1011	27.5	3.1
Stroke:			
No	3552	96.7	2.9
Yes	123	3.4	3.2
CES-D:			
No	3239	88.1	2.8
Yes	436	11.9	3.1
BMI:			
Underweight (<18.5)	46	1.25	2.4
Healthy weight (18.5–24.9)	1368	37.2	2.6
Overweight (25–29.9)	1532	41.7	3.0
Obese (>30)	729	19.8	3.3

**Table 2:** Comparison of those included and excluded from the study sample^a^.

	In study sample (N = 3675)	Not in study sample (N = 2633)	P value
	Mean (SD) or %	Mean (SD) or %	
Sex (% men)	68.4	74.0	< 0.001
Age (yr)	69.8 (5.8)	69.9 (6.0)	0.301
Ethnicity (% white)	94.2	90.5	<0.001
Employment grade (% lowest)	9.1	10.7	0.045
HCC^b^	3.5 (3.7)	6.3 (12.7)	<0.001
Original hair colour (%grey/white)	78.3	79.8	0.100
Hair dyed (% dyed)	16.5	20.8	0.004
Hair treated (% treated)	4.1	4.7	0.408
Hair washing freq. (once a week)	30.5	31.5	0.367
Season collected (% spring)	8.7	8.5	<0.001
Storage (% ≤18 months)	15.5	17.8	0.002
Steroid medication (% No)	90.9	91.4	0.544
CVD drugs	40.8	37.7	0.011
BMI% obese	19.8	19.0	0.179
Diabetes	85.7	83.8	0.035
CHD	72.5	74.0	0.187
Stroke	96.7	96.1	0.266
CES-D	88.1	85.9	0.011

a Among those eligible participants at phase 11 (N = 6308).

b Geo-means of HCC.

**Table 3:** Hair cortisol concentration by demographic and hair characteristics.

Characteristic	Unadjusted			Mutually adjusted^a^	
	Mean^b^	% difference^c^ (95% CI)	P value	% difference (95% CI)	P value
Sex:					
Male	3.80	Ref		Ref	
Female	2.90	−23.6 (−28.9, −17.8)	<0.001	−17.0 (−24.8, −8.4)	<0.001
Age groups:					
59–64	3.40	Ref		Ref	
65–74	3.55	4.4 (−3.9, 13.3.)	0.308	2.7 (−5.5, 11.6)	0.534
75–85	3.44	1.0 (−8.4, 11.4)	0.842	−2.2 (−11.9, 8.5)	0.670
Ethnicity:					
White	3.48	Ref		Ref	
South Asian	3.21	−7.9 (−22.4, 9.3)	0.344	−4.3 (−20.1, 14.6)	0.632
Black	4.44	27.3 (−9.9, 79.7)	0.171	39.6 (2.1, 99.0)	0.065
Other	4.24	21.6 (−17.7, 79.7)	0.326	32.7 (−10.2, 96.0)	0.156
Last known grade:					
Administrative	3.67	Ref		Ref	
Prof/exec	3.34	−9.0 (−15.3, −2.2)	0.010	−3.9 (−10.7, 3.5)	0.294
Clerical/support	3.19	−13.1 (−23.1, −1, −1.8)	0.025	1.1 (−11.6, 15.6)	0.871
Original hair colour:					
Grey/White	3.48	Ref		Ref	
Blonde	3.01	−13.3 (−30.0,7.3)	0.189	−0.7 (−19.9, 23.1)	0.950
Red	3.45	−0.7 (−31.2, 43.1)	0.968	6.5 (−26.0, 53.2)	0.735
Brown	3.36	−3.3 (−12.7, 7.0)	0.514	4.3 (−6.1, 15.8)	0.434
Black	4.24	22.0 (4.9, 41.9)	0.010	23.7 (5.8, 44.7)	0.008
Hair dyed:					
No	3.65	Ref		Ref	
Yes	2.76	−24.3 (−30.9, −17.1)	<0.001	−12.5 (−22.0, −1.9)	0.022
Hair treated:					
No	3.51	Ref		Ref	
Yes	2.99	−14.8 (−28.3, 1.2)	0.068	−0.6 (−17.0, 19.0)	0.945
Hair washing:					
≤Once a week	3.58	Ref		Ref	
2–4 times per week	3.42	−4.4 (−11.6, 3.5)	0.269	−3.8 (−11.3, 4.3)	0.343
≥5 times per week	3.50	−2.3 (−11.1, 7.3)	0.630	−8.4 (−17.0, 1.1)	0.081
Season collected:^d^					
Winter	4.21	Ref		Ref	
Winter-spring	3.26	−22.7 (−32.1, −12.1)	<0.001	−29.9 (−40.8, −17.0)	<0.001
Spring	2.78	−34.0(−43.2, −23.3)	<0.001	−36.0 (−46.7, −23.0)	<0.001
Spring-summer	3.25	−23.0 (−32.0, −12.9)	<0.001	−25.4 (−36.7, −12.2)	<0.001
Summer	3.42	−18.8 (−29.9, −5.9)	0.006	−20.7 (−33.9, −4.8)	0.013
Summer-autumn	3.49	17.1 (−27.4, −5.4)	0.005	−12.9 (−24.4, 0.3)	0.056
Autumn	3.83	−9.2 (−21.7,5.3)	0.200	−6.4 (−20.2, 9.8)	0.420
Autumn-winter	3.86	−8.4 (−20.0, 4.8)	0.199	−7.4 (−20.1, 7.3)	0.306
Storage:					
≤18 months	3.73	Ref		Ref	
19–24 months	3.77	1.1 (−8.5, 11.8)	0.825	−23.6 (−36.5, −8.1)	0.004
>24 months	3.14	−15.8 (−23.9, −6.9)	0.001	−18.0 (−26.1, −9.0)	<0.001

a Adjusted for sex, age, ethnicity, grade, hair colour, hair dye, hair treatment, hair washing, season, storage.

b Unadjusted geometric means.

c Percentage difference in hair cortisol concentration from the reference group.

d An eight category variable was created to take account of hair growth which during the three month sample overlapped two seasons.

**Table 4:** Hair cortisol concentration by medication and health status.

Characteristic	Unadjusted			Fully adjusted^a^	
	Mean^b^	% difference^c^ (95% CI)	P value	% difference (95% CI)	P value
Medications					
Steriods:					
No	3.50	Ref		Ref	
Yes (local)	3.72	6.2 (−6.8, 21.2)	0.367	5.8 (−7.1,20.5)	0.393
Yes (systemic)	2.17	−37.9 (−51.6, −20.4)	<0.001	−37.5 (−51.0,−20.2)	<0.001
CVD drugs:					
No	3.25	Ref		Ref	
Yes	3.66	12.5 (5.0, 20.6)	0.001	2.7 (−4.6, 10.5)	0.477

Health status					
BMI:					
Underweight	3.1	2.5 (−24.6, 39.4)	0.873	7.7 (−20.6,46.0)	0.634
Healthy weight	3.1	Ref		Ref	
Overweight	3.7	20.2 (11.4, 29.7)	<0.001	17.7 (9.0, 27.0)	<0.001
Obese	4.0	31.5 (19.7, 44.4)	<0.001	33.0 (20.7, 46.5)	<0.001
Diabetes:					
No	3.4	Ref		Ref	
Yes	4.1	21.1 (9.9, 33.4)	<0.001	14.7 (3.8, 26.8)	0.007
Coronary heart disease:					
No	3.4	Ref		Ref	
Yes	3.6	6.8 (−1.0,15.2)	0.090	1.4 (−6.2, 9.5)	0.735
Stroke:					
No	3.5	Ref		Ref	
Yes	3.9	12.5 (−6.9, 35.9)	0.222	6.0 (−12.1, 28.0)	0.541
Depressive symptoms:					
No	3.44	Ref		Ref	
Yes	3.96	15.8 (4.3, 28.6)	0.008	20.0 (8.1, 33.3)	0.001

a Adjusted for sex, age, ethnicity, grade, hair colour, hair dye, hair treatment, hair washing, season, storage steroid drugs, cvd drugs, diabetes, CHD and Stroke.

b Unadjusted geometric means.

c Percentage difference in hair cortisol concentration from the reference group.
