# Supplementary material for: Assessing cortisol from hair samples in a large observational cohort: The Whitehall II study
Source: Psychoneuroendocrinology. 2016 Nov;73:148–56. doi: 10.1016/j.psyneuen.2016.07.214 (PMC5052124; doi:10.1016/j.psyneuen.2016.07.214)
Supplement: Supplementary file 1 [file mmc1.docx]

|  | Median HCC | Mean HCC | Linear regression  Ln(HCC) and medication | |
| --- | --- | --- | --- | --- |
|  |  |  | *Beta* | *P value* |
| No medication (N=3,341) | 2.88 | 7.2 | 0.49 | <0.001 |
| “0302”  An inhaled corticosteroid (N=205) | 2.47 | 7.9 | 0.41 | 0.007 |
| “060302”  Glucocorticoid therapy (N=64) | 1.51 | 7.6 | REF | REF |
| “1304”  Topical corticosteroids (N=65) | 4.56 | 15.1 | 0.96 | <0.001 |

**Table S1: Median and Mean HCC by steroid medication type.**

**Table S2: Mean HCC, age and BMI by CHD diagnosis and CVD medication**

|  | HCC  Mean (median) | P  value | Mean Age | P  value | Mean BMI | P value |
| --- | --- | --- | --- | --- | --- | --- |
| **Diagnosed** + *CVD medication* (N=775) | 8.30 (3.11) | P=0.13 **^a^** | 72.1 | <0.001 **^b^** | 27.7 | <0.001 **^b^** |
| **Diagnosed** + No CVD medication (N=236) | 6.19 (2.86) |  | 69.0 |  | 26.4 |  |
| Not diagnosed + *CVD medication* (N=1399) | 7.80 (2.86) | P=0.005 **^a^** | 70.2 | <0.001 **^b^** | 27.4 | <0.001 **^b^** |
| Not diagnosed + No CVD medication (N=1265) | 6.56 (2.75) |  | 68.0 |  | 25.5 |  |

**^a^ Wilcoxon Rank-sum test between CVD medication /no CVD medication**

**^b^ T-test between CVD medication /no CVD medication**
